# Interaction strength promotes robustness against cascading effects in mutualistic networks

**DOI:** 10.1038/s41598-018-35803-8

**Published:** 2019-01-24

**Authors:** Marília P. Gaiarsa, Paulo R. Guimarães

**Affiliations:** Departamento de Ecologia, Universidade de São Paulo, São Paulo, SP 05508-900 Brazil

## Abstract

Perturbations, such as fluctuations in abundance, can ripple across species assemblages through ecological interactions. Furthermore, the way in which ecological interactions are organized into a network and the interaction strengths connecting species may be important for cascading effects. Previous work revealed that network structure determines how cascading effects spread across species assemblages. A next step is to understand how interaction strengths influence cascading effects. Here, we assume that perturbations have negative effects, and we evaluate whether interaction strength affects network robustness to cascading effects in mutualistic interactions, and examine the role of network structure in mediating perturbation cascades when interaction strength is incorporated. We combine empirical data on 18 mutualistic networks, two simulations scenarios, and network theory, to investigate how network structure affects perturbation spreading time, a proxy of network robustness to cascading effects. Simulations in which we included interaction strength presented higher mean spreading time, indicating that interaction strength increases network robustness. Richness, modularity, and nestedness had a strong, positive effect, on mean perturbation spreading time regardless of the interaction strengths. We found that network structure and the distribution of interaction strengths affected communities’ robustness to perturbation spreading. Our results contribute to the discussion on the danger that ecosystems face when species, and interactions alike, become extinct.

## Introduction

Perturbations, from natural disasters to anthropogenic disturbances, can reshape the structure and dynamics of ecological communities, potentially affecting a number of existing species, their abundances, and interaction patterns^1–4^. Because particular species play major roles for ecosystem functioning and the services on which society depends, perturbations may alter the functioning of ecosystems^5–7^. Given the current biodiversity crisis triggered by anthropogenic impacts such as habitat loss and climate change^6,7^, it is crucial to deepen our understanding on the robustness of ecological assemblages to the spread of perturbations^8–10^.

In ecological communities individuals of all species rely on individuals of other species to live and persist^11–13^. For example, many plants ultimately depend on the existence of their pollinators, predators on their prey, and parasites on their hosts^2,3,14^. Thus, species are linked to each other through the interactions they share, forming networks of interacting species^13^. A possible consequence of the interdependence of species within a community is the occurrence of cascading effects, defined as perturbations that spread via species interactions^2,15–19^. Cascading effects might be ubiquitous across different types of ecological assemblages, from mutualistic interactions between flowering-plant species and their pollinators^2,20^, to associations between plant species and herbivorous insects^2,21^, and between parasites and mammalian hosts^3^. Because cascading effects can potentially affect multiple species within a system^18^, it is most appropriate to focus on assemblages of interacting species in order to predict community-level responses to perturbations^9^ such as fluctuations in abundance or extinction of a single species^4^. Even though cascading effects are likely major processes shaping the ecological and evolutionary dynamics of interacting assemblages^3,22–24^, it is still unclear how robust different assemblages are to cascading effects.

It has been recently shown that the structure of ecological interactions—the way the interactions among species are organized—may affect the robustness of ecological assemblages^13,18,25^. Network structure can be described by a variety of metrics that characterize how interactions are distributed and organized among species, and several aspects of network structure have been linked to community robustness^1,8,16,26,27^. Recently, much of the work on the fragility of ecological networks has focused on connectance, modularity, and nestedness^9,28,29^.

Connectance, the proportion of interactions actually observed in relation to all possible interactions within the network^30^, has been shown to either increase^31,32^ or decrease network robustness^26^, depending of modeling assumptions. Modularity is a pattern in which species within particular subsets of the network interact more among themselves than with other species in the network^33^. On one hand, modularity may promote cascading effects within modules^34^. On the other hand, modularity may promote network robustness by limiting cascading effects among distinct modules^9,27,29^. Finally, nestedness is a pattern in which species with few interactions interact with a subset of species that also interact with highly connected species^28^. In nested networks the loss of less connected species may have little effect on network structure, rendering nested networks more robust to species loss^26,32^. However, as generalists represent the core of nested networks, losing a generalist species may have severe effects on the structure of these networks^9^, promoting cascading effects^16^.

Network structure, therefore, may have relevant consequences to the robustness of ecological networks to perturbation spreading. However, the relative importance of each ecological interaction may influence the effects of network structure. For example, Vieira *et al*.^35^ showed that interaction strengths may influence coextinctions in numerical simulations. Along the same lines, mathematical models predict that interaction strengths govern ecological stability of interacting assemblages^36,37^. Therefore, a further step to improve the current knowledge on network robustness is to explore whether species’ interaction strengths affect the dynamics of cascading effects^38^. Here, we explore how interaction strength and interaction patterns can either promote or hamper perturbation spreading in mutualistic networks. We created a stochastic model that simulates cascading effects to explore the robustness of 18 empirical, mutualistic networks. We used numerical simulations of this stochastic model to test if interaction strengths affect the spreading of cascading effects by comparing simulations in which perturbation spreading was or was not affected by the frequency of interactions between species’ pairs. We found that interaction strengths potentially hamper the rate of cascading effects, increasing the robustness of mutualistic networks.

## Materials and Methods

### Perturbation spreading model: a random Boolean approach

**Figure 1:**
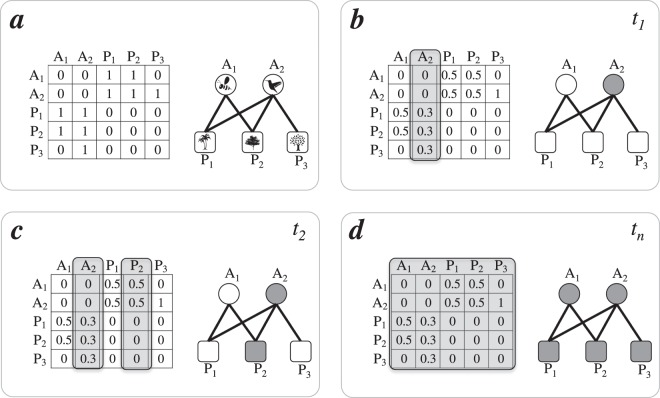
Schematic representation of the perturbation spreading model for the binary scenario. A mutualistic network composed of two animal species and three plant species can be represented as an adjacency matrix (**a**). Each element of the matrix is now divided by the total number of interaction partners each species has (species’ degree) and the simulation starts with one species (*A*_2_) being randomly selected to be perturbed (**b**). In the following time steps, the perturbed species *A*_2_ can spread the perturbation to its interaction partners (*P*_1_, *P*_2_, or *P*_3_). Species *P*_2_ is affected by the perturbation in species *A*_2_ (**c**). The simulation runs until all species are affected by the perturbation (**d**). For the quantitative scenario each element in matrix 1a is replaced by the interaction strength between that species pair (see Fig. S1).

Here, we considered perturbation in a general sense–*e.g*., the cascading effects following changes in any feature of interacting species, such as changes in species abundances^4^. We created a perturbation spreading model to investigate how perturbations may cascade in ecological networks (Fig. 1). Perturbations could be the propagation of facilitation effects across the networks but that for sake of simplicity, and because there is considerable more information on the negative effects of extinction drivers in ecological communities than on positive effects, we will focus in the scenario that perturbation may represent a negative impact. To model perturbation spreading dynamics we used a Random Boolean Network (RBN) approach^39^. In the RBN approach each node has a state, which is described by a binary variable, either equal to one (‘on’) or to zero (‘off’). The trade-off of the RBN approach is overlooking the detailed description of ecological dynamics by allowing us, in turn, to follow perturbation spreading as a qualitative effect (on/off). As a consequence, through the RBN approach we can circumvent the challenge of estimating the multiple parameters needed to describe the functional forms underlying the quantitative dynamics of species abundances. In our mutualistic networks, the nodes represent species, and the state of each node represents whether that particular species is affected by a perturbation.

At the time step *t*_0_ a perturbation affects one species, randomly chosen, *e.g*. species *A*_2_ in Fig. 1b. In the following time step all species that interact with *A*_2_ may be affected by the perturbation (*e.g*. all plant species pollinated by *A*_2_, Fig. 1c). In the RBN approach the perturbation dynamics are described by logical functions. Logical functions connect the state of a species *i* (affected by the perturbation or not) to the state of each species with which *i* interacts in a given time step. States of species *j* that interact with the perturbed species *i* are updated in each time step according to the following logical rule: perturbation spreads if two species interact and $r\le {f}_{ij}$, in which *r* is a value randomly sampled from a uniform distribution bounded between 0 and 1, and *f*_*ij*_ is the probability of a species *j* responding to a perturbation affecting an interacting partner *i*. For example, in a pollination network in which species *i* and *j* interact, a perturbation such as the decrease in the abundance of a pollinator species *i*, may lead to a decrease in the abundance of plant species *j* as a result of the perturbation in *i*.

### Considering interaction strength

To explore the role of network structure and interaction strengths we used two simulation scenarios. In the binary scenario the probability of a species being affected by the perturbation, *b*_*ij*_, was given by:

1 $${b}_{ij}=\frac{1}{{k}_{i}}$$

in which *k*_*i*_ is the number of species interacting with species *i*, that is, the degree of *i* (Fig. 1b). Thus, we assume that the more *k* partners *i* has, the less it depends on *j* and, therefore, more unlikely it is for species *i* to be affected by a perturbation on species *j* (Fig. 1). Similarly, in the quantitative scenario, we explored how pairwise interaction frequencies affect mean perturbation spreading time (Fig. S1). In the quantitative scenario the probability *l*_*ij*_ of a species being affected by the perturbation was defined as:

2 $${l}_{ij}=\frac{{q}_{ij}}{\sum _{k=1}^{R}{q}_{kj}}$$

where *q*_*ij*_ and *q*_*kj*_ represent the frequency of interactions (*i.e*., number of events) involving individuals of species *i* and *j* and of species *i* and *k*. For example, in pollination networks this represents the number of times a pollinator species was seen interacting with a plant species, and in seed-dispersal networks the number of times an animal was seen consuming or dispersing fruits. Again, we assumed that the greater the interaction strength, the more likely it is for a species to be affected by a perturbation in one of its partners. The comparison between binary (no effect of interaction strength) and quantitative scenarios (effects of interaction strength) allows us to explore how the inclusion of interaction strength affects cascading dynamics.

Perturbations may propagate through the network without leading species to extinction. Also, even if a species dies out, the effects of its extinction may be long-lasting. To simulate this broad class of phenomena, unlike in the seminal studies by Dunne *et al*.^31^ and Memmot *et al*.^15^, we did not remove species in our simulations. Hence, in both scenarios, the probability of a species being affected by the perturbation (*b*_*ij*_ and *l*_*ij*_) is constant, since species’ interaction patterns remain unaltered through time. Thus, network structure remains constant through time, allowing us to explore the relationship between network structure, interaction strengths, and cascading effects.

For each scenario we characterized simulation dynamics as the number of species affected at a given time step, and produced a sequence of dynamic states until all species in the network were affected by the perturbation (Fig. 1d). In nature, cascading effects may disappear before the entire community is affected. However, our aim is not to derive a prediction to the time of collapse of a given network but to use this model to evaluate network fragility: if mean spreading time of a cascading effect is small, then one may infer that the network favors cascading effects in our simulations, being less robust to perturbation spreading. In contrast, if mean spreading time is large, then the network structure buffers cascading effects in our simulations.

For each network and for each scenario, we performed 1000 simulations. A sensitivity analysis supported that 1000 simulations was enough to reach asymptotic estimates of the mean spreading time (Fig. S2). Since all networks in our dataset are connected, *i.e*., there is a path of interactions connecting directly or indirectly all pairs of species in the network, the perturbation spreads throughout the entire network. Our response variable represents how many time steps were necessary for all the species in the network to be affected by the perturbation. We then calculated mean perturbation spreading time across all 1000 simulations and defined robustness as how many time steps it takes for a perturbation to spread across the network, affecting all species.

We used a paired t-test to compare if there was a difference in mean perturbation spreading time between the binary and the quantitative scenarios, using the mean spreading time for 1000 simulations in each scenario in a given empirical network as a paired sample. To explore how network structure may either facilitate or hamper perturbation spreading, we performed a path analysis^40,41^ between mean perturbation spreading time and four structural metrics—richness, connectance, nestedness, and modularity (see below). We assumed that richness and connectance have a direct effect on mean spreading time^31,32^, that richness and connectance affect nestedness^28^ and modularity^33^, all of which also have a direct effect on mean spreading time (Fig. S3). We evaluated the entire causal structure of the path model simultaneously^40,41^, for both scenarios (binary and quantitative) separately.

### Mutualistic networks

Our dataset comprises 18 empirical networks of mutualistic interactions (nine plant-seed disperser and nine plant-pollinator; Table S1) that capture a range of structural patterns and network properties observed in seed dispersal and pollination assemblages^42,43^. To compute network metrics and perturbation spreading time for each empirical network, we used an adjacency matrix (**A**) and a quantitative matrix (**Q**) for each network (Fig. S1).

The adjacency matrix, **A**, describes the occurrence of interactions between *N* animals and *P* plant species. In **A**, each element *a*_*ij*_ represents the interaction between animal *i*, depicted as a column of **A**, and plant *j*, depicted as a row of **A**. Thus, when two species interact *a*_*ij*_ = 1, or *a*_*ij*_ = 0 otherwise (Fig. 1a). The quantitative matrix **Q** provides information on interaction strengths, defined here as the number *q*_*ij*_ of interaction events between any given species’ pairs (Fig. S1). Greater numbers of interaction events imply greater interaction strengths, and higher values of *q*_*ij*_. The matrices **Q** and **A** are related in such a way that if ${q}_{ij} > 0$ then *a*_*ij*_ = 1; otherwise *q*_*ij*_ = *a*_*ij*_ = 0.

### Descriptors of network structure

We used four metrics to characterize structural properties of networks: (*i*) species richness, R, (*ii*) connectance^30^, (*iii*) nestedness^28,44^, and (*iv*) modularity^33^. Species richness is simply the total number of species within the network. Connectance^30^ is the proportion of all possible interactions between species that are realized, and it is calculated as *C* = *I*/*NP*, in which *I* is the total number of interactions in the network, *N* the total number of animals and *P* the total number of plants. Nestedness^28^ was calculated using the NODF^45^, that ranges from 0 (not nested) to 100 (perfectly nested). Modularity values range from 0 to 1 and were estimated through the metric *M*^33,46^ with the software MODULAR^47^.

## Results

**Figure 2:**
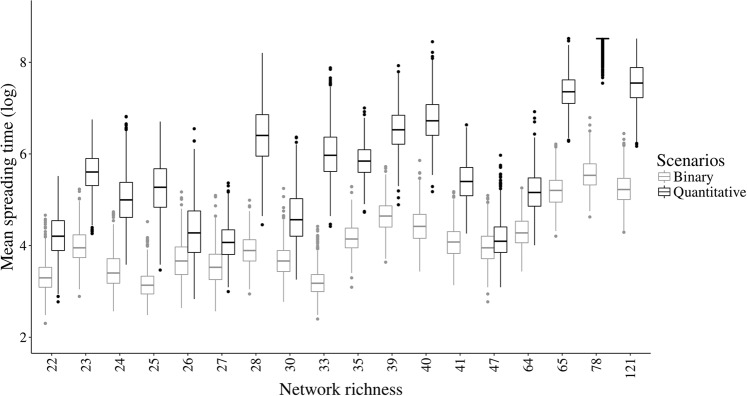
Mean spreading time and standard deviation (log) for all networks analyzed, ordered by increasing richness.

**Figure 3:**
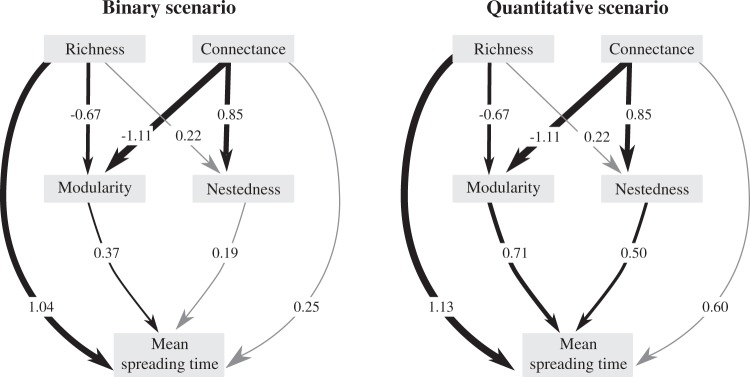
Summary diagram of the path analysis of the different network architectural patterns on mean spreading time analyzed for the binary and the quantitative scenario. Arrow thickness is scaled to standardized coefficients from the path analysis and illustrates the relative effect strength. Significant effects are represented in black lines and non-significant effects are represented in gray lines. Effects of connectance and richness are split between direct effects and indirect effects through changes in modularity and nestedness.

Mean spreading time varied greatly between the binary and the quantitative scenarios (Figs. 2 and S4; Tables S2 and S3), for each of the 18 networks analyzed. In all networks perturbation spreading time was smaller in the binary scenario than in the quantitative scenario (paired *t*_(18)_ = −2, 57, *p* = 0.02; Figs 2 and S4; Tables S4 and S5). When we consider each scenario separately, the relationship between the analysed structural metrics and mean spreading time showed similar results (Figs. 3, S5, and S6, Tables S4 and S5). In both scenarios richness had the largest, positive effect on mean spreading time and connectance had a non-significant effect (Fig. 3). Mean spreading time in the binary scenario was mostly affected by richness (1.04) and modularity (0.37; Fig. 3; Table S4). Similarly, mean spreading time in the quantitative scenario was mostly affected by richness (1.13) and modularity (0.71), but it was also affected by nestedness (0.50; Fig. 3, Table S5). The overall effects of richness and connectance in modularity and nestedness remained the same between scenarios, since these metrics are independent of interaction strength (Fig. 3, Tables S4 and S5).

## Discussion

We show that interaction strengths and network structure are related to network robustness to perturbation spreading. We assumed that perturbations had negative effects and show that the inclusion of interaction strengths increases network robustness, since perturbations take longer to affect all species in the network than when we consider only the presence/absence of interactions (our binary scenario). Furthermore, in both scenarios, richness had a strong, positive effect on mean spreading time, indicating that networks with more species are more robust to perturbation spreading than networks with smaller richness. Contrary to what other theoretical approaches predict^16,25,31^, we found no effect of connectance on network robustness to perturbation spreading. However, both modularity and nestedness had a positive effect on mean spreading time, indicating that more modular and nested networks will be robust to cascading effects. If we consider that perturbations may also have positive outcomes, in networks with greater richness, modularity, and nestedness, the effects we found would be more likely to be amplified, leading to cascading effects that may spread positive effects. Positive effects, in turn, may generate unstable effects, such as fluctuation of population densities and traits.

Theoretical studies of cascading effects in mutualistic systems have so far considered the number of secondary extinctions that follows species removal^15,16^, or explored network robustness to a particular structural metric, such as nestedness^48^. Similarly, Vieira *et al*.^35^ investigated the robustness of mutualistic networks based on species dependence on the mutualistic interaction. To our knowledge, however this is the first study to explore the effects of interaction strength on the robustness of mutualistic networks to perturbation spreading when network structure remains unaltered. Interaction strength in mutualistic networks is highly asymmetric, with only a few species presenting strong interactions in each network^49,50^. We provide further evidence that this asymmetry in interaction strength confers robustness to mutualistic networks^25,50^ because all networks were more robust to perturbation spreading in our quantitative scenario. Moreover, our results suggest that studies that only consider interaction presence (our binary approach) to evaluate community robustness to cascading effects might be overestimating the speed with which these perturbations propagate in real communities.

In both scenarios, perturbations took longer to affect all species in mutualistic networks with greater species richness than in species-poor networks. The effect of species richness on community robustness and stability has long been debated in ecological literature^1,51^, with contrasting results, partly due to the different types of perturbation and the different robustness definition considered^19,52^. Here, we defined robustness as how long it takes for all species in a network to be affected by a perturbation. Our results indicate that richer communities are more robust to perturbation spreading than communities with a smaller number of species supporting the biodiversity insurance hypothesis^19,52–54^. It is also noteworthy that richness had a stronger, direct, effect on mean spreading time in the quantitative scenario than in the binary scenario (Fig. 3). Thus, we can hypothesize that as species become extinct and richness decreases, the robustness of ecological communities to perturbation spreading will also decrease. However, even though we found a strong, positive relationship between robustness and mean spreading time, this relationship is not trivial since networks with greater richness not always showed a greater mean spreading time (Fig. 2).

Besides richness, the way in which the different interactions are organized within the network may be linked to network robustness to cascading effects. We found a strong, positive effect of modularity and nestedness on mean spreading time. Thus, our results are in line with the existing literature on modularity, nestedness, and robustness of ecological networks that suggest that high levels of modularity and nestedness might render networks more robust^16,25,26,29,32^. Therefore, in more nested and/or more modular networks perturbations took longer to spread than in less modular networks, rendering these networks more robust to perturbation spreading. Thereby, we provide aditional evidence that modules may act as buffers against perturbations, restricting the perturbation spreading to within specific modules^29^.

We show that there is a clear relationship between network structure and the robustness of communities to perturbation spreading. Our results suggest that ecological communities will become increasingly vulnerable to cascading effects, as species are lost, richness decreases, and biodiversity is eroded. Furthermore, Vidal *et al*.^55^ demonstrated that species that contributed the most to network organization were also the most endangered species. Thus, in addition to negatively affecting the structure of interaction networks, species extinction may cause communities to become more prone to adverse cascading effects.

## Electronic supplementary material

SUPPLEMENTARY INFO
