# Supplementary material for: Interaction strength promotes robustness against cascading effects in mutualistic networks
Source: Sci Rep. 2019 Jan 24;9:676. doi: 10.1038/s41598-018-35803-8 (PMC6345762; doi:10.1038/s41598-018-35803-8)
Supplement: Supplementary file 1 — SUPPLEMENTARY INFO [file 41598_2018_35803_MOESM1_ESM.pdf]

Supplementary material

Table S1: Analyzed networks and topological metrics. Type: S=seed-dispersal, P=pollination. Please refer to text for more information.

**Network**	**Type**	**Richness**	**Connectance**	**Nestedness**	**Modularity**
Baird (1980)	S	28	0.34	50.98	0.32
Beehler (1983)	S	40	0.43	67.66	0.22
Bezerra et al. (2009)	P	26	0.42	84.93	0.23
Carlo et al. (2003)	S	41	0.17	44.7	0.4
Dicks et al. (2002)	P	78	0.14	52.27	0.4
Galetti & Pizo (1996)	S	64	0.14	35.49	0.38
Jordano (1985)	S	33	0.44	78.76	0.3
Mosquin & Martin (1967)	P	30	0.23	42.72	0.39
Olesen et al. (2002) (Aigrettes)	P	27	0.29	51.87	0.34
Olesen et al. (2002) (Flores)	P	22	0.25	35.96	0.44
Ollerton et al. (2003)	P	65	0.2	35.49	0.44
Poulin et al. (1999) (Micwgt)	S	23	0.43	48.29	0.35
Poulin et al. (1999) (Pscwgt)	S	24	0.37	73.9	0.26
Schemske et al. (1978)	P	39	0.26	53.4	0.36
Schleuning et al. (2011)	S	121	0.14	34.59	0.31
Small (1976)	P	47	0.32	40.96	0.27
Sorensen (1981)	S	25	0.3	45.92	0.33
Vazquez & Simberloff (2003)	P	35	0.22	30.3	0.49

Table S2: Results for the binary scenario. Mean spreading time (Mean), maximum spreading time (Max), minimum spreading time (Min), and standard deviation of spreading time (SD) for each of the analyzed networks.

**Network**	**Mean**	**Max**	**Min**	**SD**
Baird (1980)	52.56	147	19	18.82
Beehler (1983)	90.41	350	31	37.27
Bezerra et al. (2009)	43.49	176	14	20.05
Carlo et al. (2003)	63.04	177	23	22.61
Dicks et al. (2002)	271.88	891	102	94.15
Galetti & Pizo (1996)	77.98	192	31	28.07
Jordano (1985)	25.87	83	11	8.88
Mosquin & Martin (1967)	41.92	190	16	16.3
Olesen et al. (2002) (Aigrettes)	37.85	163	13	16.9
Olesen et al. (2002) (Flores)	29.44	106	10	11.51
Ollerton et al. (2003)	192.42	500	67	67.6
Poulin et al. (1999) (Micwgt)	57.25	187	18	21.93
Poulin et al. (1999) (Pscwgt)	34.26	114	13	15.35
Schemske et al. (1978)	110.57	306	38	39.2
Schleuning et al. (2011)	202.12	630	73	76.44
Small (1976)	56.45	163	16	21.8
Sorensen (1981)	24.84	92	12	8.18
Vazquez & Simberloff (2003)	67.74	198	22	22.31

Table S3: Results for the quantitative scenario. Mean spreading time (Mean), maximum spreading time (Max), minimum spreading time (Min), and standard deviation of spreading time (SD) for each of the analyzed networks.

**Network**	**Mean**	**Max**	**Min**	**SD**
Baird (1980)	735.92	3657	86	496.5
Beehler (1983)	958.5	4666	177	526.12
Bezerra et al. (2009)	93.42	699	17	69.47
Carlo et al. (2003)	244.78	761	71	112.65
Dicks et al. (2002)	4746.02	5000	1889	571.97
Galetti & Pizo (1996)	199.84	1014	55	103.22
Jordano (1985)	473.1	2640	83	304.39
Mosquin & Martin (1967)	120.71	582	26	77.18
Olesen et al. (2002) (Aigrettes)	64.39	214	20	27.01
Olesen et al. (2002) (Flores)	77.26	249	16	40.31
Ollerton et al. (2003)	1686.65	5000	532	657.55
Poulin et al. (1999) (Micwgt)	300.65	856	71	137.6
Poulin et al. (1999) (Pscwgt)	175.04	913	36	109.35
Schemske et al. (1978)	759.05	2770	133	364.84
Schleuning et al. (2011)	2122.34	5000	477	1018.94
Small (1976)	70.75	392	22	38.89
Sorensen (1981)	224.53	817	32	130.75
Vazquez & Simberloff (2003)	369.72	1099	113	138.11

Table S4: Summary of the path analysis coefficients for the binary scenario (mean = mean spreading time).

		**Estimate**	**Std.Err**	**z-value**	**P(>—z—)**	**Std.lv**	**Std.all**
Mean	~						
Richness	(e)	1.571	0.311	5.050	0.000	1.571	1.041
Modularity	(m)	1.179	0.568	2.077	0.038	1.179	0.373
Nestedness	(n)	0.464	0.379	1.223	0.222	0.464	0.194
Connectance	(f)	0.459	0.530	0.867	0.386	0.459	0.252
Modularity	~						
Richness	(j)	-0.317	0.103	-3.084	0.002	-0.317	-0.665
Connectance	(h)	-0.638	0.124	-5.143	0	-0.638	-1.109
Nestedness	~						
Richness	(g)	0.141	0.154	0.917	0.359	0.141	0.224
Connectance	(k)	0.644	0.186	3.471	0.001	0.644	0.846

Table S5: Summary of the path analysis coefficients for the quantitative scenario (mean = mean spreading time).

		**Estimate**	**Std.Err**	**z-value**	**P(>—z—)**	**Std.lv**	**Std.all**
Mean	~						
Richness	(e)	3.255	0.656	4.962	0	3.255	1.131
Modularity	(m)	4.302	1.197	3.593	0	4.302	0.713
Nestedness	(n)	2.288	0.800	2.859	0.004	2.288	0.502
Connectance	(f)	2.064	1.117	1.848	0.065	2.064	0.594
Modularity	~						
Richness	(j)	-0.317	0.103	-3.084	0.002	-0.317	-0.665
Connectance	(h)	-0.638	0.124	-5.143	0	-0.638	-1.109
Nestedness	~						
Richness	(g)	0.141	0.154	0.917	0.359	0.141	0.224
Connectance	(k)	0.644	0.186	3.471	0.001	0.644	0.846

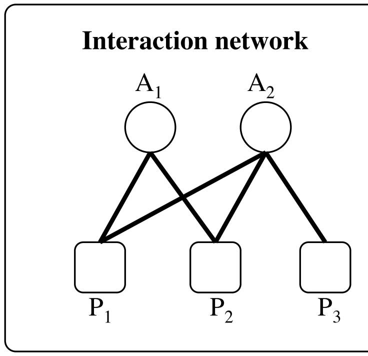

	P_1	P_2	P_3
A_1	1	1	0
A_2	1	1	1

	P_1	P_2	P_3
A_1	7	1	0
A_2	10	3	7

Figure S1: Schematic representation of the matrices used in this study. The interaction network can be represented as the adjacency matrix **A**, which depicts if two species interact or do not interact. The quantitative matrix **Q** provides information on the number of interaction events between species pairs.

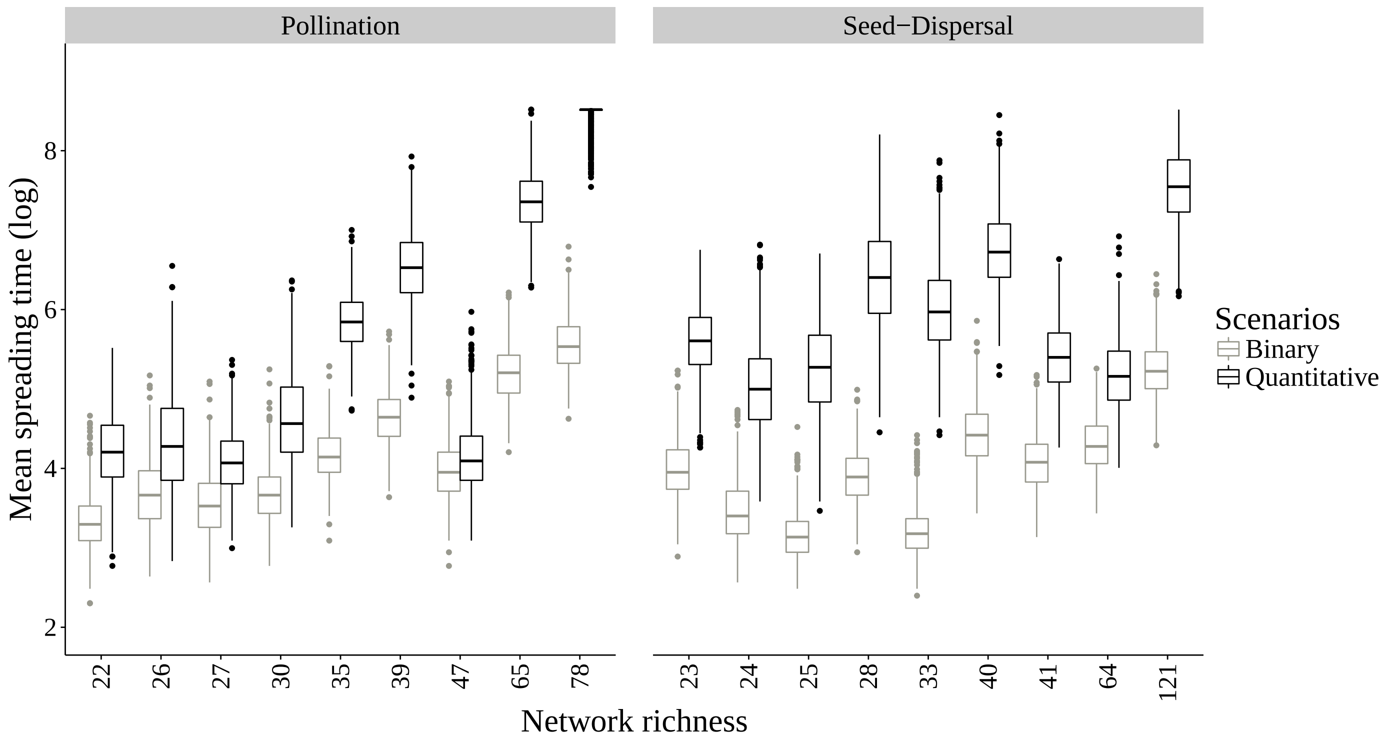

Figure S2: Mean spreading time (log) and standard deviation for the binary (in grey) and the quantitative (in black) scenarios, separated by pollination and seed dispersal networks and ordered by increasing richness.

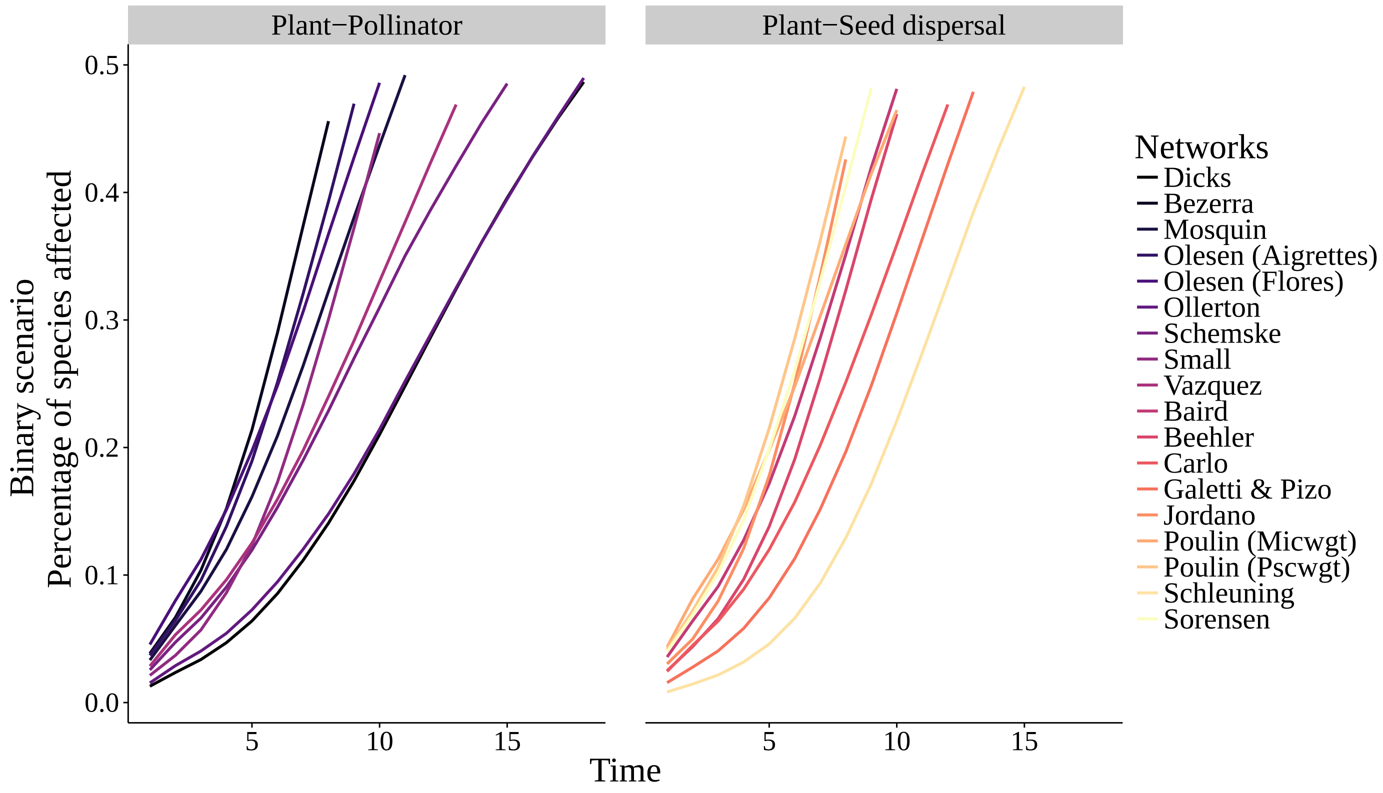

Figure S3: Percentage of species affected (up to 50%) at each time step, for each network type in the binary scenario. Each line represents a different network, and there was no difference between mean spreading time and network type (paired $t(13.7) = 0.74, p = 0.47$).

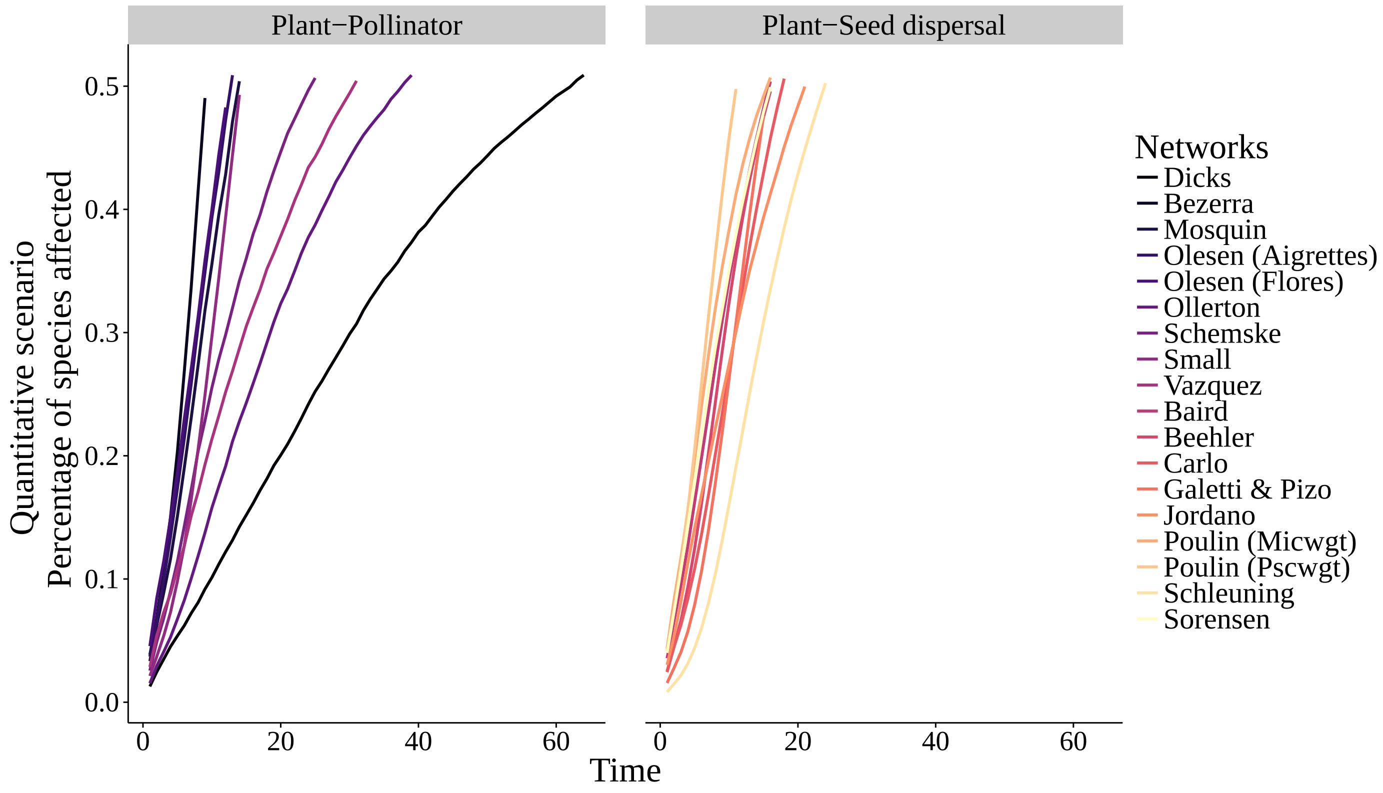

Figure S4: Percentage of species affected (up to 50%) at each time step, for each network type in the quantitative scenario. Each line represents a different network, and there was no difference between mean spreading time and network type (paired $t(10.6) = 0.51, p = 0.62$).

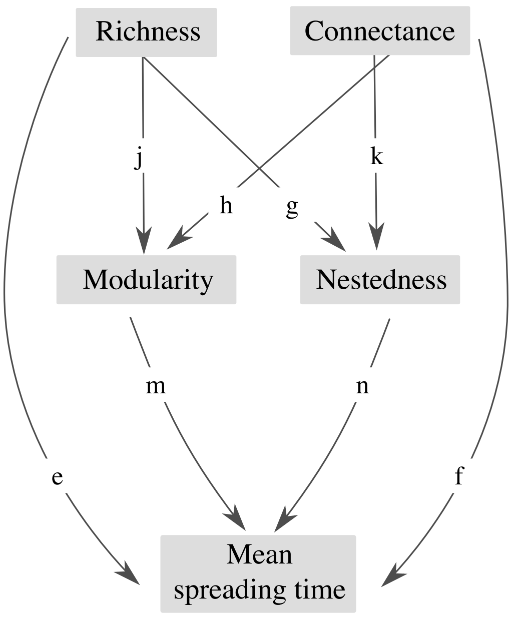

Figure S5: Relationship among variables used in path analysis.

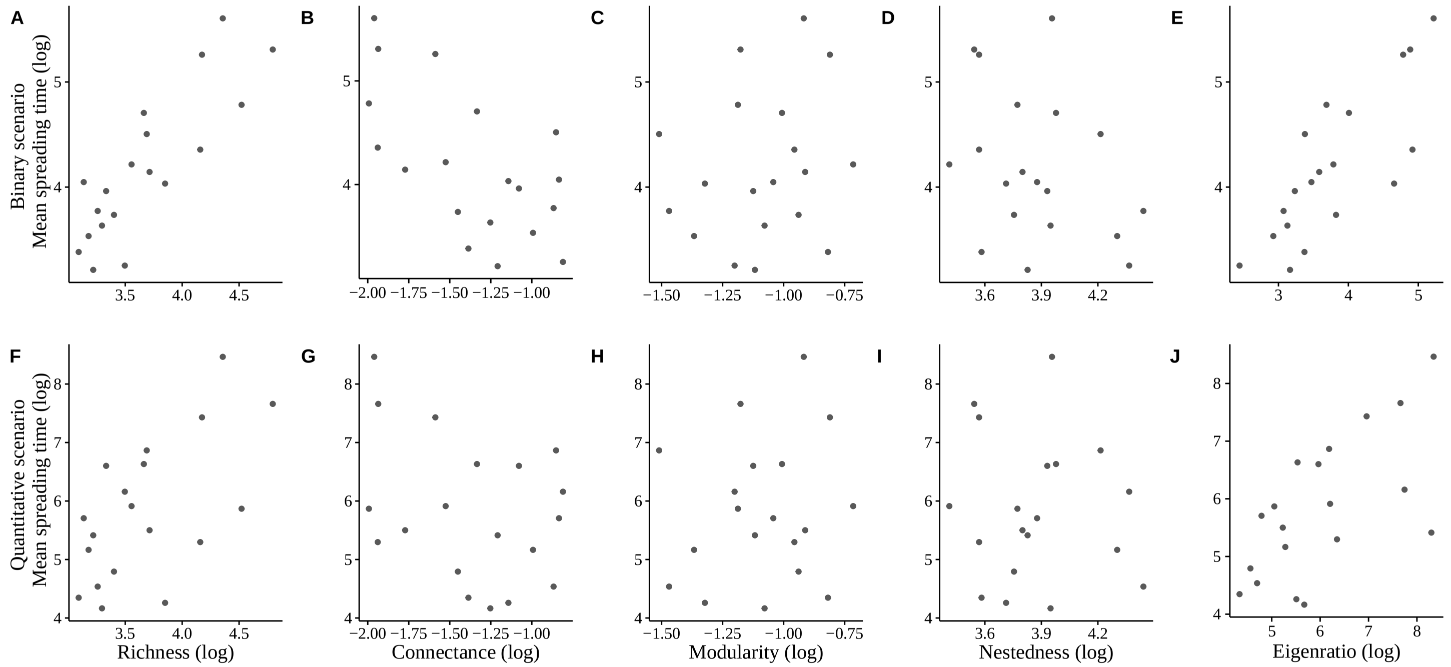

Figure S6: Correlation between analyzed metrics and mean spreading time in the binary and quantitative scenarios. Due to the high correlation between mean spreading time and richness in the binary scenario (A), for the figures B, C, and D, we plotted the residuals of the linear regression between mean spreading time and richness (refer to text for more information).
